# Healthcare Management and the Humanities: An Invitation to Dialogue

**DOI:** 10.3390/ijerph18136771

**Published:** 2021-06-24

**Authors:** Nathan Gerard

**Affiliations:** Department of Health Care Administration, California State University, Long Beach, CA 90840, USA; nathan.gerard@csulb.edu

**Keywords:** humanities, healthcare management, interdisciplinarity, professionalization, vulnerability, anxiety

## Abstract

Throughout the fields of medicine and organization studies, there are growing indications of the value of the humanities for enriching scholarship, education, and practice. However, the field of healthcare management has yet to consider the promise of the humanities for illuminating its particular domain. This perspective paper explores how the humanities might begin to play a role in healthcare management by focusing on three broad areas: (1) understanding the lived experiences of management, (2) offsetting the “tyranny of metrics”, and (3) confronting rather than avoiding anxiety. While preliminary in presentation, these areas are intended to facilitate wider consideration of the humanities in healthcare management and to encourage interdisciplinary dialogue. The paper also identifies actionable approaches that might be derived from such a dialogue, including substantiating critical healthcare management scholarship, collaborating with humanities educators to design novel curricula, proposing alternatives to unduly circumscribed performance targets and competency assessments, creating case studies of formative experiences of practicing healthcare managers, and advancing guidelines for better managing anxiety and its concomitant stress, burnout, and compassion fatigue in healthcare organizations. The paper concludes by discussing the potential risks of incorporating the humanities into healthcare management, while also offering a prospective synthesis from an interdisciplinary approach.

## 1. Introduction

Barely a century old [1,2,3], the field of healthcare management has matured into a global presence [4,5], boasting an extensive knowledge base of increasingly data-driven and evidence-based approaches [6,7,8,9]. However, in the midst of such approaches, healthcare managers must continue to channel their intuition and exercise judgment, demonstrate critical and creative thinking, and cultivate a tolerance for ambiguity [10,11,12]—all skills best honed, this paper argues, through a sustained engagement with the humanities. Building on efforts to incorporate the humanities into the fields of organization studies [13,14,15], medicine [16,17], and other allied health professions [18,19,20,21], this paper seeks to demonstrate how the humanities might begin to play a role in healthcare management by focusing on three broad areas: (1) understanding the lived experiences of management, (2) offsetting the “tyranny of metrics” [22], and (3) confronting rather than avoiding anxiety. Hardly exhaustive, these areas are intended to foster recognition that both the humanities and healthcare management share a basic desire to understand a complex human world. Furthermore, actional approaches that derive from these three broad areas are offered to demonstrate how scholars and practitioners of healthcare management might take steps toward more interdisciplinary research, pedagogy, and practice.

Before proceeding, a few caveats are in order. First, to argue for the value of the humanities in healthcare management is not to argue against data-driven or evidence-based knowledge, or more generally against science. Rather, science and technology are not abandoned by the humanities but placed in a broader and more nuanced context. At the same time, the humanities should not be confused with efforts to mobilize science and technology to better humanity or “the greater good,” however much such efforts may align with insights derived from the humanities. “Literature is not an instrument of social change or an instrument of social reform,” notes the late literary critic Harold Bloom. “It is more a mode of human sensations and impressions, which do not reduce very well to societal rules or forms” [23] (p. 326). There are, of course, counterpoints to Bloom that emphasize the role of the humanities in buttressing cultural and moral values and fostering global citizenship (particularly those made by Nussbaum [24] and Eagleton [25]), but the general consensus remains that the humanities cannot be easily distilled into formulaic prescriptions. Second, and related, incorporating the humanities into healthcare management does not entail simply doing qualitative research or examining literature and philosophy for testable hypotheses. Instead, the humanities should be encountered on their own terms—including their tendency to stir up strong emotions, show rather than tell, and more generally plumb the depths of human nature in a manner that invites a unique and sustained form of reflection. It is this distinct value of the humanities that weaves through each of the three broad areas explored below.

## 2. Understanding the Lived Experiences of Management

Scholars and practitioners of healthcare management have long recognized the inadequacy of traditional academic silos for addressing a complex and rapidly changing industry [26]. However, precisely because of its expansive scholarly terrain, healthcare management is a field with no disciplinary “home”. Residing tenuously at the crossroads of business, public policy, health science, and medicine (to name a few), healthcare management struggles to locate itself in the broader ecosystem of academia [27]. In an intriguing parallel, the humanities—once the intellectual heart of the university and the center of liberal arts education—have also experienced a recent homelessness of sorts, displaced by tectonic shifts rippling through not just academia but the whole of contemporary society [28]. The difference, of course, is that healthcare management is often perceived as part of the reason for the humanities’ demise, poaching students who might otherwise pursue an interest in philosophy, literature, or the arts with the lure of a so-called “job-ready” degree [29]. Such institutional antagonism is unfortunate as it eclipses an opportunity for finding common ground and particularly for discovering within the humanities an opportunity to reflect on a more general precarity in the lived experiences of healthcare management—both for those being managed and those having to manage—amid powerful organizational and societal forces. In other words, through “lived experiences”, the humanities may help to illuminate the immediate, first-hand encounters of healthcare managers that are often ignored or obscured by more formalized descriptions of the field.

Take, for instance, a recent offshoot of medical humanities known as “health humanities”, which has given rise to interdisciplinary programs geared toward undergraduates interested in various health careers [30]. As Klugman notes, such programs “provide baccalaureate students with an inoculation against medical culture that often does not value the human. Medical schools subliminally de-emphasize patient interaction, communication, and reflective reason. Patients’ decisions are often influenced by advertising and physician recommendations…studying the humanities as baccalaureate students may provide a vaccination against these biases” [31] (p. 426). While arguably a provocative statement, Krugman’s framing of the subliminal influence of medical schools calls into question the influence of healthcare management programs and, specifically, whether a similar vaccination effect might manifest as a result of a “hidden curriculum” [32] of business education, which, as critical healthcare management scholars point out [33], implicitly teaches students how to wield power through exclusionary “expert” knowledge, often in direct proportion to a declining interest in social and political issues. “With education in humanities,” notes Örtenblad, “we are all better prepared to be aware of the ongoing indoctrination and standardization of us and our minds, and to reflect on and criticize these instead of just following them” [34] (p. 293). Or stated in less extreme, more inviting terms, with education in the humanities, we may find greater value in the practice of reflexivity and questioning often overlooked implicit assumptions, and through this, appreciate the susceptibility of our minds to unconscious influence.

In a related sense, healthcare management might find within the humanities a set of shared interests that call into question efforts to “professionalize” the field through standardized knowledge and prescribed training [35]. As Noordegraaf and van der Meulen have noted in this context, “professionalization is no neutral phenomenon, because big personal, financial and organizational stakes are at play, and because political and professional circumstances offer many ambiguities” [36] (p. 1068). Because of this, the authors conclude that “professionalization will have to be seen as ‘projects’ that are full of ‘power play’”, and this risks devolving into “homogenizing forms of control” at the expense of fostering the more critical and creative aspects of the field [36] (pp. 1058, 1068). Seen from the vantage point of the humanities, however, professionalization might serve as platform for discovering new meaning for healthcare management [37]. For starters, humanities scholars have pointed out that the actual practice of management is far from standardized, scripted, or even at times professional, but instead often messy and uncertain [38]. However, this need not mean, as critics may argue, that the field is somehow corrupt and in need of “unmasking” [11]. Rather, “managers need, in other words, some knowledge of human characters; of human potential and of human foibles,” notes Hendry [14] (p. 277), in order to bring their own complicated realities to life. Furthermore, standardized knowledge and prescribed training could be complemented by the experience-near knowledge of the humanities, which in turn may shed novel light on the quest for professional power. As McClay notes, “the humanities attempt to understand the human condition from the inside” [39] (p. 34), which, in the case of healthcare management, entails understanding the insecurities and anxieties of healthcare managers that may compel the need for homogenizing forms of control.

It is worth noting that in the medical humanities, John Berger’s now-classic *A Fortunate Man: The Story of a Country Doctor* [40], offers precisely this knowledge for students and practitioners of medicine. Heralded as a “masterpiece of witness: a moving meditation of humanity, society, and the value of healing” [41], Berger’s book succeeds in conveying what conventional medical school curricula simply cannot—namely, to use Berger’s own words, “the extraordinary complex convergence of philosophical traditions, feelings, half-realized ideas, atavistic instincts, and imaginative intimations, which lie beneath the simplest hope or disappointment of the simplest person” [40] (p. 110). For the healthcare manager, an equivalent book has yet to be written, presenting an untapped opportunity for the field to benefit from the sustained witnessing of a novelist like Berger. Through such witnessing, healthcare management might foster a different kind of professional sovereignty; one rooted less in the need to secure status and legitimacy and more in appreciating the “extraordinary complex convergence” of meaning that confers the field’s unique sense of social purpose [40].

To further expand upon the promise of the humanities for understanding the lived experiences of healthcare management, scholars and practitioners might consider the following actions. First, for those who identify with the growing sub-field known as “critical healthcare management studies” [42], the humanities can deepen the practice of critique by grounding it in what Hendry calls “knowledge of human characters” [14] (p. 277), making critique not just more accessible but also more powerful. Second, educators stand to benefit from collaborating with their colleagues in the humanities to explore co-teaching opportunities and novel curricula that inculcates a more robust questioning of norms and implicit assumptions, a greater appreciation for entering the experiential world of the other, and, above all, a deeper sensitivity to one’s own lived experiences. For example, in the related field of global health, Stewart has recently “experimented with bringing together humanities, arts, and global health faculty, plus their students, to stimulate interdisciplinary conversation, inspire reflections on student motivations to study global health, and prompt critiques of global health practices and programming” [43] (p. 10), even going so far as to outline a set of preliminary class assignments. While it is perhaps premature to circumscribe precisely where the humanities could be infused into existing healthcare management pedagogy, some more established guideposts can be found in the medical humanities, which offer a range of touchpoints across medical school curricula, up to and including specialized concentrations, certificates, and minor degrees [44].

## 3. Offsetting the “Tyranny of Metrics”

In his recent book, *The Tyranny of Metrics*, Jerry Muller [22] reveals how so-called “metric fixation” has come to consume organizations—and especially healthcare organizations—threatening our livelihoods and the health of our institutions. Muller traces this fixation back to the rarely questioned assumption that standardized measurement, expressed in numeric form, proves far superior to any personal and therefore “subjective” judgment; an assumption that then fuels the belief that effective management equates to what can be measured, while the un-measurable becomes irrelevant.

Subjective judgement, of course—and almost by definition—is difficult to teach. But the humanities offer as good a pedagogical platform as any to discover the meaning of what cannot be measured in healthcare management. Indeed, nowhere is this arguably more needed than in the field’s push to establish and measure professional competencies [45,46], an initiative spearheaded by accreditation agencies that increasingly dictate the pedagogical content of healthcare management programs. For the critic, there is a deep irony here: healthcare, often ridiculed for its bureaucratic tendencies [47], has succumbed to reinforcing a bureaucracy of thought. For the humanities scholar, however, there is an occasion to broaden the scope of competencies to include those cultivated through mediums such as literature and poetry. Empathy, for example, which lies at root of truly compassionate care, requires that one actually embody the feeling of another [48], a skill difficult to measure short of obfuscation through Likert-type assessments, yet something the complexity of characters portrayed in literature may help to foster. As Michaelson, notes, “only a novel, with its omniscient access to characters’ actions and inner states, could recondition our ethical encounters with fictional worlds to lead us empathetically to rethink those real-world phenomena that we encounter only from outside” [15] (p. 2).

Similarly, encounters with fictional worlds encourage perspective-taking, a skill that proves elusive to measure by virtue of its shifting and open-ended presentation. However, scholars working at the intersection of the humanities and organization studies have turned to poetry, and particularly to John Keats’ [49] idea of “negative capability”, to describe poetry’s potential for cultivating perspective-taking in leaders [50,51]. For Keats, negative capability is synonymous with “being in uncertainties, Mysteries, doubts, without an irritable reaching after fact or reason” [49] (p. 43), whereas for Simpson et al., it entails “the capacity to sustain reflective inaction” including “waiting, observing, and listening” in the face of the common pressure thrust upon leaders to act [51] (pp. 1210–1211).

Taken together, both empathy and perspective-taking involve the passing of time coupled with a tolerance for suspense. The narrative or character arc of a novel or poem cannot be rushed without distorting the plot, and the arc itself does not easily track to a steady progression of development or the accumulation of measurable abilities suggested by competency model assessments. In other words, the image of the individual as a project to be constantly worked on and improved upon—what Cremin has called “the ongoing reflection on the self as object of exchange, a kind of ongoing self-assessment” [52] (p. 45)—is itself exchanged for a deeper form of self-enrichment. As two pioneers [53,54] in the emerging area known as “applied organizational poetry” convey, “poetry does not answer questions. It allows for and creates space in which ambivalence and ambiguity are protected, even encouraged—without threat of punishment—to arise, to be tried on, and in so doing, provisional answers to life experience may emerge” [53].

Some actionable approaches that derive from valuing subjective judgment, empathy, and perspective-taking as fostered by the humanities include making space to reconsider the use of metrics in healthcare management, and perhaps most pressingly the use of pay-for-performance programs. As Roland and Campbell have noted in the UK context, pay-for-performance that rewards only what can be measured (such as mortality rates) leads to “less holistic care and inappropriate concentration of the doctor’s gaze on what can be measured rather than what is important” [55] (p. 1947)—or in other words, “treating to the test.” The uncanny parallel with “teaching to the test” (or to measurable competencies) is noteworthy here as a phenomenon that leaves both educators and students hamstrung into a way of learning that precludes patience and a tolerance for what cannot be measured. Of course, the humanities are not the sole force to counteract pay-for-performance risks, nor more generally those of “metric fixation,” but their emphasis on “waiting, observing, and listening,” as Simpson et al. note, puts the healthcare manager “in the place where new knowledge may emerge” [51] (p. 254). However, “when the pressure is on… to meet targets based exclusively on the measurement of outputs, the ‘default’ position tends to be *control*. In conditions such as these it seems inevitable that this ‘negative’ capacity will tend to be ignored or to pass unnoticed, and will atrophy by being excluded from dominant organizational discourses” [51] (p. 254; italics in original). Similarly, the humanities might cultivate a tolerance for uncertainty, complexity, and being present, all of which are not only vital to navigating an ever-changing healthcare environment but also to finding meaning and purpose in one’s work beyond the mere achievement of measurable abilities or pay-for-performance targets. Specifically, what this might look like in practice can be gleaned from action research conducted by Lips-Wiersma and Morris [56], which “actively involved participants in the process of affirming and uncovering the meaningfulness of their work” through insights from the humanities. Through interactive workshops that employed art and poetry, a total of 216 participants across various occupations were engaged in an experience of meaning-making that offered “maximal space to uncover and express personal meaning” [56] (p. 499). As the authors observe, “participants had clearly articulated beliefs that coming to terms with an imperfect self in an imperfect world is of existential significance” and that “work is considered to be more meaningful when one can be aware of imperfections, not knowing” [56] (p. 506).

## 4. Confronting Rather Than Avoiding Anxiety

The tyranny of metrics suggests a more general “need to know” or feel in possession of knowledge, so as not to appear ignorant. To demonstrate measurable competence, in other words, is therefore to distance oneself from the charge of incompetence, which is arguably a fear that motivates the proliferation of standardized assessments [37]. Yet, accessing our incompetence may be precisely what is needed in the face of overwhelming suffering—a reality the current COVID-19 pandemic has made all too real.

Without diminishing the extraordinary heroism displayed by both clinicians and managers during this time, the pandemic has shown “that decisions made and actions taken by all manner of organizations have profound effects on human suffering or thriving” [57] (p. 2). Additionally, over the past year, we have often witnessed not just breakdowns in managing care but also how the old wounds of racial injustice and poverty resurface and intersect with healthcare organizations. In the US, where the author resides, for example, Black people are dying from COVID-19 at a rate 2.4 times higher than White people, suggesting (among other things) unequal access to quality healthcare and insurance coverage [58]. While long overdue conversations about the field’s complicity with systemic oppression are just beginning [59], the humanities have yet to be considered as a platform to facilitate such a dialogue in way that brings humility, nuance, and psychological depth. Moreover, the humanities might counteract the common tendency to resort to polarizing rhetoric that rapidly swings between defensive protectionism of the status quo and calls to “tear the system down.”

First, it would be foolish to deny that many people in healthcare management are driven by a desire to fundamentally change how society manages care. However, these same individuals become bogged down by various occupational demands, leaving them with little energy to pursue the higher callings that initially brought them to the field. However, underneath such “managerial fatigue” is perhaps a more subtle yet institutionally entrenched demand to avoid rather than confront the anxiety stirred up by giving, receiving, and managing care [60,61]. Returning again to fiction, Ken Kesey’s *One Flew Over the Cuckoo’s Nest* [62] offers a profound commentary not just on society’s mistreatment of the mentally ill, for which it is famous, but also on the way anxiety is managed and ultimately mismanaged in healthcare settings, with devasting repercussions for patients. In the film adaptation of Kesey’s book, the hospital administrator Dr. Spivey is actually played by a real psychiatrist and superintendent of the Oregon State Hospital, Dr. Dean Brooks, who served as an advisor on the film and was later cast into the role. Curiously, while Dr. Spivey’s administrative distance is often seen as tangential to the overarching plot—at best, an “ineffectual bureaucrat” who enables cruelty by leaving patients “to the mercy of repressive heavies like Big Nurse Ratched” [63]—Brooks himself was a longtime advocate for community-based services, seeing in Kesey’s book an “allegory about institutions” and particularly the way a debilitating system compromises one’s managerial responsibility [64]. The story, in other words, is not just a testament to how power circulates in healthcare and its effect on who the system serves [65,66], but also how institutions defend against anxiety in the face of madness—both outside and within.

In a more contemporary exploration of these themes, the play “Lipstick Lobotomy”, composed by Krista Knight [67], examines the cross section of gender, medical treatment, and politics. Through exposing not just the professional overreach in implementing questionable treatments but also the patient’s desperation for relief, the play wrestles with the common tension between the desire to care and the desire to cure. It is the latter desire that, like the lobotomy procedure itself, all too easily becomes a way to aggressively purge anxiety at the expense of the more difficult and ongoing task of caring—for oneself as much as for others.

In addition to captivating their audience, both stories point to how the humanities might function to put healthcare managers back in touch with their own “story”—whether that be the calling propelling them into the profession, the vulnerability that joins them with those in need, or the capacity to absorb the shocks of the pandemic and persevere. In the interdisciplinary approach known as narrative medicine, for instance, which is now infused into the curricula of many prestigious medical schools, the practices of close reading and reflective writing help to validate the experiences not just of marginalized patients and caregivers (due to structural inequalities and social injustice) but also the physicians themselves [68]. Extended to healthcare management, these practices might go some way in challenging the entrenched view of the healthcare manager (and the organization he or she oversees) as a disembodied entity [57], free from anxiety, replacing this with a more capacious view that includes hope, despair, and resilience. Of course, this is not to discount the pressure that many in healthcare management may feel to retain some distance from their anxiety and meet expectations for appearing competent. However, this pressure need not coincide—as it so often does—with the questionable assumption that detachment and dispassion are the hallmarks of rigorous research or professional practice. In the broadest sense, the humanities offer a different set of assumptions about what a human is and what a human could be, challenging scholars and practitioners of healthcare management to reflect on their own assumptions with curiosity, courage, and a deeper sense of care.

In sum, confronting rather than avoiding anxiety with the aid of the humanities suggests the following implications for interdisciplinary research, pedagogy, and practice. First, works of literature, theater, and the arts are replete with stories of courage in the face of anxiety that do justice both to the sense of internal crisis and paralyzing fear that can accompany anxiety, as well as to the meaning and direction that can be derived from working through rather than avoiding it. Translative links to healthcare management contexts are needed, such as in the form of case studies of crisis moments in leaders, that further draw connections between insights from the humanities and real-world phenomena. In addition, as the above examples suggest, the humanities provide a rich stage for exploring the consequences of avoiding anxiety, not just from a personal (or existential) but also a moral and political perspective. To effectively confront anxiety, therefore, entails far more than mere individual-level coping skills; rather, organizations and institutions share an obligation to create containing spaces where anxiety can be felt, explored, and worked through as a basic facet of our shared humanity. Such a seemingly simple obligation carries the potential to transform the practice of healthcare management yet arguably goes unheeded, as rising levels of stress, burnout, and compassion fatigue among healthcare workers can attest [69,70].

## 5. Conclusions

This paper is rooted in the premise that the humanities—a cluster of academic disciplines spanning over 2000 years and comprising some of civilization’s deepest thoughts on society, culture, and the human condition—have something valuable to contribute to the field of healthcare management. However, this paper only begins to touch upon such an interdisciplinary endeavor, leaving ample room for more targeted explorations of the field’s intersection with specific disciplines, such as literature, philosophy, poetry, and the arts. Moreover, like any interdisciplinary endeavor, there are potential risks. First, the dialogue between healthcare management and the humanities could become too intellectualized, exclusive, and thus exclusory for those who may be intrigued but do not claim expertise in a particular discipline of study. Not only would this be at odds with the ethos of this paper, it would also risk positioning the humanities as yet one more specialty to add to a growing curriculum or to include on a syllabus, draining the humanities of their experiential value. Likewise, the humanities may risk becoming deployed as merely an academic lever of critique for those disenchanted with the field, which further alienates scholars and practitioners who to wish to discover ways “to affirm what they are already doing and to help them to do it better” [71] (p. 34). The alternative risk, however, is that in bringing the humanities to a new audience and context they become distorted—or worse, watered down—with humanities scholars crying foul for such misappropriation.

While these potential risks are considerable, they should not foreclose on the opportunities afforded by interdisciplinary dialogue. In the interim, perhaps the biggest barrier to overcome is the assumption that the humanities only focus on abstract, fanciful, or lofty ideas while the field of healthcare management only focuses on this-worldly problems. With the aid of the humanities, scholars and practitioners of healthcare management might renew their field with a sense of wonder and even awe. Healthcare organizations, after all, when managed well, offer some of the few havens where miracles can still take place in a secular world. Moreover, the humanities might do justice to the incredible depth of feeling that can accompany illness and suffering, as well recovery and hope, allowing healthcare managers to make links between their daily tasks and the humans beings who are profoundly affected by them. The humanities, in turn, might find within healthcare management a renewed sense of purpose, offering a rich well of ideas and forms of expression to a new domain that in turn reasserts the humanities’ distinct value. As McClay rightly observes, “we need the humanities in order to understand more fully what it means to be human, and to permit that knowledge to shape and nourish the way we live” [39] (p. 35).

## Data Availability

Not applicable.
